# An accurate prognostic staging system for hepatocellular carcinoma patients after curative hepatectomy

**DOI:** 10.3892/ijo.2014.2798

**Published:** 2014-12-17

**Authors:** YUKIO TOKUMITSU, TAKAO TAMESA, SATOSHI MATSUKUMA, NORIAKI HASHIMOTO, YOSHINARI MAEDA, YOSHIHIRO TOKUHISA, KAZUHIKO SAKAMOTO, TOMIO UENO, SHOICHI HAZAMA, HIROYUKI OGIHARA, YUSUKE FUJITA, YOSHIHIKO HAMAMOTO, MASAAKI OKA, NORIO IIZUKA

**Affiliations:** 1 Department of Digestive Surgery and Surgical Oncology, Yamaguchi University Graduate School of Medicine, Yamaguchi, Japan; 2 Department of Biomolecular Engineering Applied Molecular Bioscience, Yamaguchi University Graduate School of Medicine, Yamaguchi, Japan; 3 Yamaguchi University, Yamaguchi, Japan

**Keywords:** hepatocellular carcinoma, recurrence, staging system, prognostic factor, Akaike information criterion

## Abstract

The aim of this study was to develop an accurate predictive system for prognosis of hepatocellular carcinoma (HCC) patients after hepatectomy. We pooled data of clinicopathological features of 234 HCC patients who underwent curative hepatectomy. On the basis of the pooled data, we established a simple predictive staging system (PS score) scored by the mathematical product of tumor number and size, and degree of liver function. We compared the prognostic abilities of the PS score (score 0–3) with those of six well-known clinical staging systems. Then, we found that there were significant differences (P<0.05) in both disease-free survival (DFS) and overall survival (OS) between patients with different PS scores (PS score 0 vs. 1; PS score 1 vs. 2), and there was a significant difference in DFS, but not OS, between patients with PS score 2 and those with PS score 3. Moreover, the PS score had smaller values of the Akaike information criterion for both DFS and OS than any of the six well-known clinical staging systems. These results suggest that the PS score serves as a simple, accurate predictor for the prognosis of HCC patients after hepatectomy.

## Introduction

Hepatocellular carcinoma (HCC) is one of the most common malignancies worldwide. Liver resection for HCC has the highest local controllability among all local treatments; however, the recurrence rates of HCC remain high even after curative hepatectomy (1–3). Because of the high recurrence rate and poor prognosis, the prognostic assessment and selection of treatment strategy in HCC patients are quite important (4,5). In particular, a precise stratification system for the prognosis of HCC patients is required in parallel to the advent of effective systemic treatment options (6).

It is well known that the prognosis of HCC depends on both tumor factors (i.e., size and extent of primary tumor) and host factors (i.e., liver function) (7); however, the latter is not integrated in the tumor lymph node metastasis (TNM) staging system, which is generally accepted as a standard approach for prognostication in many cancer clinical staging systems. Even when primary HCC is completely treated, recurrence is observed much more frequently in the form of multicentric carcinogenesis in the residual cirrhotic liver, because the potential for multicentric carcinogenesis increases with the progression of chronic liver disease and liver cirrhosis (2,3). Therefore, from the standpoint of prognosis, a staging system based on information regarding both tumor factors and host factors such as liver function are required to accurately classify HCC patients undergoing various therapeutic options. For this reason, many prognostic staging systems for HCC, such as the Japan Integrated Staging score (JIS score), modified JIS score, the Cancer of the Liver Italian Program (CLIP) score, and the Tokyo score, have been proposed during the last two decades (8–11). However, the prognostic ability of these systems cannot be universally accepted because of the variation in cohorts and/or subjects. It remains unclear which of the staging systems is most accurate for predicting the prognosis of HCC. Therefore, in the present study, we developed a new staging system that had high accuracy in predicting the recurrence of HCC by driving data for factors used in the known staging systems.

## Patients and methods

### Patients

We pooled data on the clinicopathological features of 304 patients who underwent curative hepatectomy for HCC at Yamaguchi University School of Medicine between 1985 and 2009. The diagnoses of HCC were all confirmed pathologically. We defined curative hepatectomy as complete resection of all tumor nodules without involving any major branch of the portal or hepatic veins. We excluded 29 patients who had follow-up periods <5 years, and excluded 41 patients who had undergone treatment options such as percutaneous ethanol injection therapy (PEIT), microwave coagulation therapy (MCT), radiofrequency ablation (RFA), or transcatheter arterial chemoembolization (TACE) prior to surgery. Ultimately, 234 patients were enrolled in the study.

Data on tumor factors such as size of the main tumor, number of tumors, tumor differentiation, and vascular invasion were based on the final pathological findings of the resected liver. Laboratory data, including albumin, bilirubin, prothrombin activity, platelet count, indocyanine green retention rate at 15 min (ICG-R15), α-fetoprotein (AFP), and positivity for viral markers (hepatitis B surface antigen and anti-hepatitis C antibody), were obtained before operation. The Child-Pugh classification (12), the degree of Liver Damage classification by the Liver Cancer Study Group of Japan (LCSGJ) (13), TNM staging system (LCSGJ), TNM staging system (Union for International Cancer Control, UICC), JIS score, modified JIS score, CLIP score, and Tokyo score were evaluated using these variables. All patients were followed up after hepatectomy until death or the date of last follow-up visit, and survival was censored in December 2013.

### Development of predictive staging system (PS score)

We attempted to identify tumor factors that were closely related to the prognosis of HCC after surgery. Subsequently, we constructed a novel multidimensional staging system by combing the identified tumor factor and some host factors.

By analyzing the variables and their combinations that were used in the TNM staging system (data not shown), we found that the mathematical product (NxS factor) of tumor number and size (cm) had high accuracy in predicting recurrence of HCC. We next determined the optimal cut-off values of the NxS factor at 4 and 9 in reference to the Milan criteria (single tumor ≤5 cm in size or ≤3 tumors each ≤3 cm in size) (14). The cut-off points of the NxS factor at 4 and 9 classified the 234 patients into 3 groups. All HCC patients with NxS factor <4 were within the Milan criteria and had a low incidence of recurrence. Almost all patients with NxS factor >9 were outside the Milan criteria and had a high recurrence rate. The remaining HCC patients with NxS factor 4–9 were considered to be at intermediate risk for recurrence of HCC (data not shown).

Among the host factors used in four staging systems (JIS score, m-JIS score, CLIP score, and Tokyo score), we searched for the factors with the highest prognostic probability in the postoperative clinical course of HCC patients in combination with the NxS factor.

### Statistics

Disease-free survival (DFS) and overall survival (OS) curves were plotted with the Kaplan-Meier method. Differences in DFS and OS between the groups were compared by using a log-rank test in univariate analysis. Variables that had statistical significance (P<0.05) for DFS in the univariate analysis were subsequently entered into a multivariate Cox proportional hazards model. Then, we established a new prognostic scoring system (PS score) by using the combination of factors that retained significance in multivariate analysis.

To compare the prognostic ability of each staging system, the Akaike information criterion (AIC) (15) within a Cox proportional hazards regression model was used as a measure of relative goodness-of-fit. The AIC statistic was defined by AIC = −2 log likelihood + 2 × the number of parameters in the model and −2 log likelihood calculated using the Cox model. Therefore, a smaller AIC value indicates a more desirable model for predicting outcome (9,10,16–18). For all tests, P<0.05 was considered significant. Statistical analysis was performed using JMP version 9.0 (SAS Institute Japan, Tokyo, Japan) and the Statistical Package for Social Sciences version 11 (SPSS Japan, Tokyo, Japan).

## Results

### Patient characteristics

The baseline characteristics of the 234 HCC patients are shown in Table I. The median age of the patients was 63.2±0.57 [mean ± standard error (SE)] years. Among them, 187 patients (79.9%) were males and 47 patients (20.1%) were females. There were 146 patients who were positive for antibodies to the hepatitis C virus (HCV), 44 patients positive for hepatitis B surface (HBS) antigen, and 44 patients negative for both HCV antibody and HBS antigen. In the present study, 206 and 28 patients were judged as A and B of the Child-Pugh classification, respectively. There were 148 and 86 patients who were judged as A and B of the degree of Liver Damage classification proposed by the Liver Cancer Study Group of Japan, respectively. In the present study, there were no patients classified with Child-Pugh C or Liver Damage C.

### HCC recurrence and survival rates

Among the 234 patients, 164 experienced recurrence of HCC within 5 years after hepatectomy. The disease-free median survival time was 23.2 months [95% confidence interval (CI), 20.1 to 29.7 months], and the 1-, 3- and 5-year DFS rates were 75.6, 39.7 and 29.9%, respectively. The type of recurrence was mainly intrahepatic [143 (87.2%) of 164 cases], not extrahepatic. Among the 164 cases, 12 (7.3%) had both extrahepatic and intrahepatic recurrences, and 9 (5.5%) had extrahepatic recurrence without intrahepatic recurrence.

**Figure 1:**
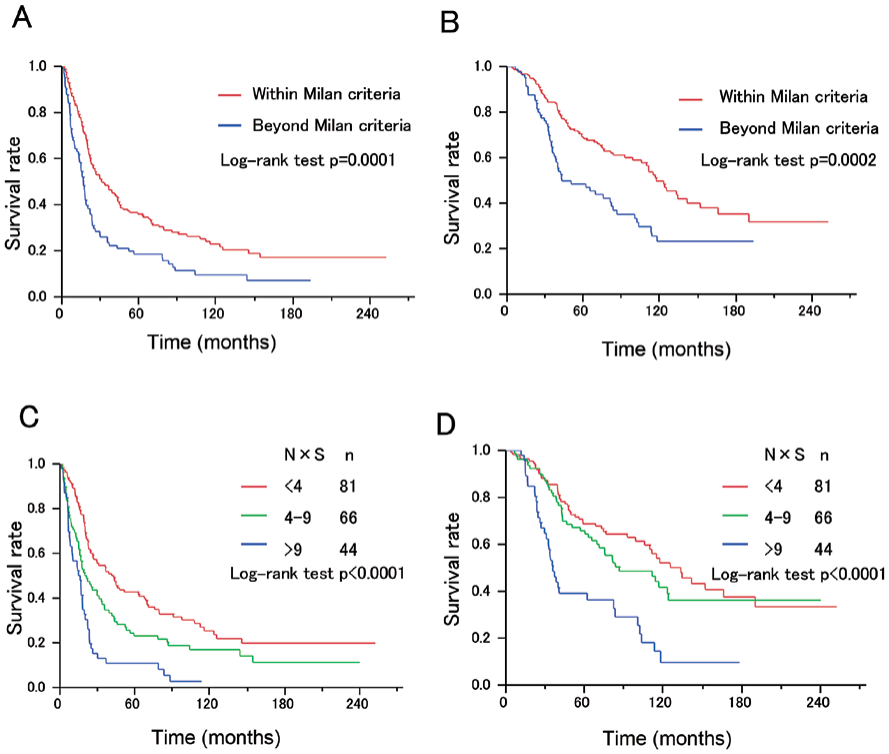
(A) Disease-free survival curves of patients according to the Milan criteria. (B) Overall survival curves of patients according to the Milan criteria. (C) Disease-free survival curves of patients according to the PS stage. (D) Overall survival curves of patients according to the PS stage.

There were statistical differences in DFS and OS between the two groups divided according to whether they fit the Milan criteria (Fig. 1A and B). The 1-year DFS rates were 88.2, 69.2 and 56.5%, the 3-year DFS rates were 54.6, 34.6 and 13.0%, and the 5-year DFS rates were 42.7, 23.1 and 10.9% in patients with NxS factor <4, NxS factor 4–9 and NxS factor >9, respectively (Fig. 1C and D). There were statistical differences in DFS and OS among the groups classified by NxS factor (P<0.0001) (Fig. 1C and D).

Log-rank analysis for DFS identified NxS factor, microscopic portal vein invasion, microscopic hepatic vein invasion, Child-Pugh classification, and degree of Liver Damage as significant prognostic factors (Table II). DFS was not associated with tumor differentiation and serum AFP levels. Multivariate analysis of the five variables that achieved statistical significance in the univariate analysis for DFS revealed that NxS factor and the degree of Liver Damage classification were independent predictors (Table II). Notably, the hazard ratio (HR) of the degree of Liver Damage classification showed higher values (HR 1.93; 95%CI 1.37–2.70; P=0.0002) in comparison with other host factors.

### PS score

Given that the NxS factor and the degree of Liver Damage classification were independent risk factors for HCC prognosis by multivariate analysis, we constructed the PS score by combining the NxS factor with the degree of Liver Damage classification. The score for the NxS factor can be easily obtained by allocating NxS factor <4, 4–9 and >9 to Liver Damage scores 0, 1 and 2, respectively (Table III). The score for the degree of Liver Damage classification can be similarly obtained by allocating Liver Damage A and B to scores 0 and 1, respectively (Table III).

**Figure 2:**
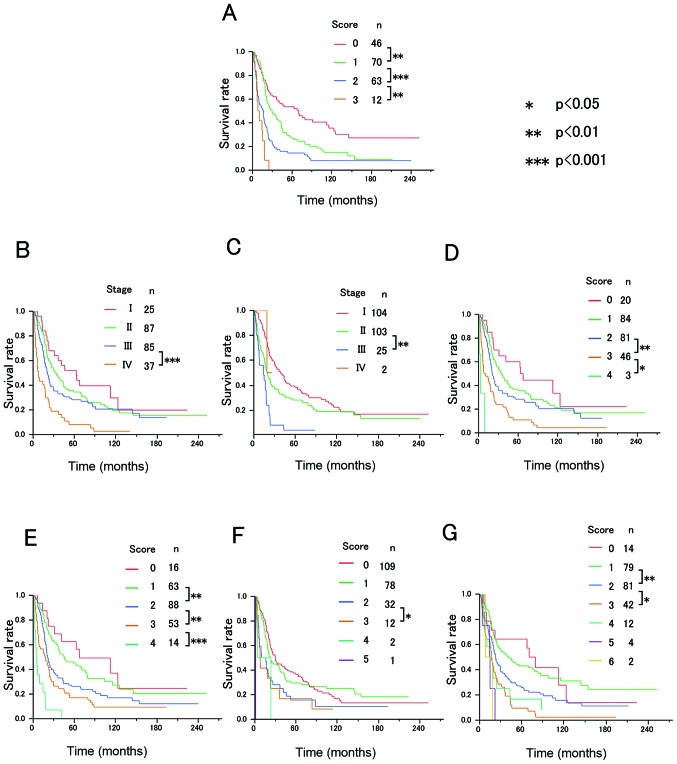
Comparison of disease-free survival according to scoring systems: (A) PS score, (B) TNM (LCSGJ), (C) TNM (UICC), (D) JIS, (E) M-JIS, (F) CLIP, and (G) Tokyo score. TNM, tumor lymph node metastasis; LCSGJ, Liver Cancer Study Group of Japan; UICC, Union for International Cancer Control; JIS, Japan Integrated Staging score; m-JIS, modified Japan Integrated Staging score; CLIP, Cancer of the Liver Italian Program.

**Figure 3:**
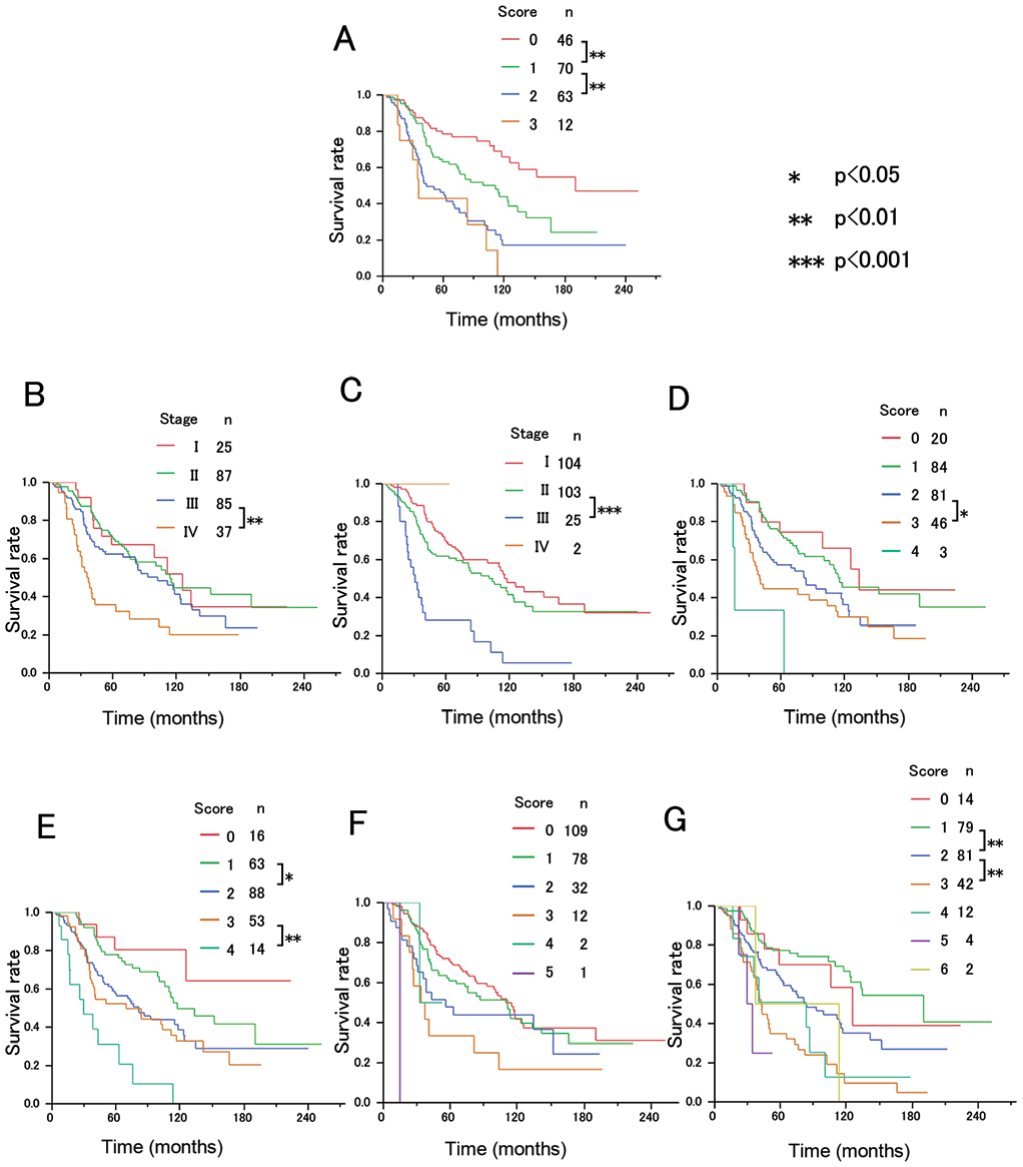
Comparison of overall survival according to scoring systems: (A) PS score, (B) TNM (LCSGJ), (C) TNM (UICC), (D) JIS, (E) M-JIS, (F) CLIP, and (G) Tokyo score. TNM, tumor lymph node metastasis; LCSGJ, Liver Cancer Study Group of Japan; UICC, Union for International Cancer Control; JIS, Japan Integrated Staging score; m-JIS, modified Japan Integrated Staging score; CLIP, Cancer of the Liver Italian Program.

The Kaplan-Meier estimated DFS curves and OS curves according to PS score, TNM staging system (LCSGJ), TNM staging system (UICC), JIS score, modified JIS score, CLIP score, and Tokyo score are depicted in Figs. 2 and 3, respectively. There were significant differences (P<0.05) in both DFS and OS between patients with different PS scores (PS score 0 vs. 1; PS score 1 vs. 2), and there was a significant difference in DFS, but not OS, between patients with PS score 2 and those with PS score 3 (Figs. 2 and 3). Among the seven staging systems, only the PS score demonstrated significant differences in DFS between all adjacent strata.

### Assessment of predictive ability of staging systems

The PS score had smaller values of the Akaike information criterion for both DFS and OS than any of the six clinical staging systems [the TNM staging system (LSCGJ), TNM staging system (UICC), CLIP score, JIS score, m-JIS score and Tokyo score] (Table IV).

## Discussion

We have developed a novel clinical scoring system, the PS score, for accurate prediction of the outcome of HCC patients after surgery by combining tumor factors and liver function. The most important findings of the present study were that there were significant differences in DFS between HCC patients with different PS scores (score 0 vs 1; score 1 vs. 2; score 2 vs. 3), and the PS score had smaller values of the AIC for DFS than any of the six well-known clinical staging systems. Such a precise stratification for DFS in HCC patients after surgery will provide a better understanding of the metastatic potentials of HCC, which will become more and more essential in parallel with the advent of effective systemic treatment options including molecular-targeted agents (6).

Our successful result was largely due to the integration of tumor number and the size of the largest tumor into the NxS factor; tumor number and size of largest tumor were each used as the main parameters in the TNM staging systems. The Milan criteria (14) hinted that we could increase the predictive power of the outcome of HCC patients if we applied the Milan criteria to our system. We found that the NxS factor had high accuracy in predicting HCC recurrence by setting the optimal two cut-off values based on the Milan criteria. Notably, the NxS factor can be obtained from modalities such as computed tomography (CT) or magnetic resonance imaging (MRI). Therefore, our newly-developed PS score is an easy-to-use preoperative assessment tool because the information on pathological vessel involvement is not needed, which is one of the parameters for the conventional TNM staging systems and is integrated into the JIS score and the modified JIS score (7,16,19).

Unsuccessful preoperative assessment causes a potential limitation in these clinical staging systems due to discrepancy between the pre- and postoperative status of HCC patients (19). In this regard, the PS score is fascinating because the score can be determined preoperatively via several imaging modalities. HCC patients have various backgrounds and divergent clinical courses, resulting in the lower predictive power of many clinical staging systems (20–22). In particular, liver function is considered to be important from the viewpoint of multicentric carcinogenesis and a necessity of additive surgical therapies (23,24).

Recently, several staging systems for HCC based on information of both tumor factors and liver function have been proposed. The CLIP score has been successful in discriminating HCC patients with regard to OS (11,25) and has been well validated in predicting the prognosis of HCC patients (26–28). Nevertheless, the CLIP score functioned poorly in predicting OS and DFS in Japanese HCC patients enrolled in the present study (Figs. 2F and 3F). One possible explanation of this deficiency is that the criteria of 50% for tumor extension in the liver used in the CLIP score system failed to accurately capture features of HCC in Japan, where many smaller tumors are detected based on the established screening system for HCC.

To overcome the deficiency of the CLIP score, Kudo *et al* developed the JIS score, composed of the Child-Pugh classification and the TNM staging system of LCSGJ. Kudo *et al* reported that this system had superior prognostic capabilities regarding OS in HCC patients when compared with the CLIP score (10,29). Thereafter, the JIS score gradually came to be used for the evaluation of treatment modalities in Japan.

In the present study, the JIS score worked poorly in predicting DFS and OS of HCC patients undergoing hepatic resection because most of them had Child-Pugh A disease. There was less advantage with the JIS score, which was made by combining two systems, the Child-Pugh classification and the TNM staging system of the LCSGJ (8,16). Therefore, many Japanese liver surgeons have not considered the JIS score to be effective in HCC patients who have undergone hepatic resection (30).

More recently, Nanashima *et al* developed the m-JIS score, which was constructed using the degree of Liver Damage and the TNM staging system of the LCSGJ. These investigators showed that the m-JIS score was a better predictor of prognosis than the JIS score in HCC patients who underwent hepatic resection (8,16). Ikai *et al* validated this system using the records of 42,269 HCC patients (31). In the present study, the m-JIS score demonstrated better stratification for prognosis after surgery and a smaller AIC value than did the JIS score. This finding is supported by the concept that the assessment of the degree of Liver Damage classification in the m-JIS score, in which ICG-R15 was substituted for encephalopathy in the Child-Pugh classification, could evaluate and classify liver function more precisely than the Child-Pugh classification.

ICG-R15 (representing indocyanine green clearance) has been used in the field of surgery routinely in Japan as a useful marker of hepatic function and as a gauge for decision-making regarding the permitted volume of hepatic resection (32,33). Moreover, ICG-R15 is a significant prognostic factor in HCC patients (34–36). The substitution of ICG-R15 for encephalopathy in the PS score might have reflected individual differences more accurately among patients who underwent hepatic resection in the present study, because none of them had any encephalopathy before operation.

In addition, the PS score had a smaller AIC value than did the m-JIS score with respect to both DFS and OS. This finding is supported by the concept that the NxS factor was more prognostic than TNM staging by the LCSGJ for tumor characteristics. The difference between the PS score and the m-JIS was confined to the tumor factor because the degree of Liver Damage was incorporated in both the PS score and the m-JIS score as liver function.

The Tokyo score is a new prognostic scoring system for patients who are candidates for radical therapy, such as percutaneous ablation or surgical resection (9). This system consists of four factors: tumor size, number of tumor nodules, serum albumin and bilirubin. These factors can be obtained from laboratory data or images before surgery. Although the Tokyo score can also become a clinical staging system for HCC patients to predict their prognosis before operation, this system has demonstrated poorer stratification than did the PS score in the present study. The Tokyo score may have been especially limited in its ability to stratify patients with an advanced score (Tokyo scores 4–6) in the present study, because most patients who had advanced cancer with poor liver function in this study were not subjected to hepatic resection.

In the present study, we were unable to evaluate the Barcelona Clinic Liver Cancer (BCLC) staging system (37), Chinese University Prognostic Index (38), or other recently reported systems (17, 39), because portal pressure, performance status, and alkaline phosphatase levels were not fully recorded for the patients enrolled in the present study. Although portal hypertension is accepted as a strong predictor of poor prognosis (40,41), formal measurement of hepatic venous pressure gradient is a special examination that is not routinely carried out in daily practice. However, all of the parameters used by the PS score can be easily and safely obtained before the operation.

In conclusion, the present study showed that the PS score was superior to any of the well-known systems, including CLIP, JIS, modified-JIS, and the Tokyo score in predicting the prognosis of HCC patients.

There were several limitations in the present study: it was a retrospective single-center study that enrolled only patients who underwent curative hepatectomy. In this regard, further studies will be needed to evaluate whether the robustness of the PS score in predicting prognosis could be maintained in a cohort in which the majority of the subjects were HCC patients who received non-surgical treatment.

## Figures and Tables

**Table I:** Patient profiles and tumor characteristics.

Variable	No. of patients
Host factors	
Age	
Mean ± standard error (years)	63.2±0.57
Gender	
Male/female	187/47
Viral infection	
HBV/HCV/non-BC	44/146/44
Platelet count (x10^3^/mm^3^)	
<10/≥10	62/172
Degree of liver damage	
A/B	148/86
Child-Pugh classification	
A/B	206/28
Tumor factors	
Tumor size (cm) (maximum diameter)	
<2/≥2, <5/≥5	35/153/46
Number of tumors	
1/2–3/≥4	155/64/15
The product of NxS factor	
<4/4–9/>9	110/78/46
Macroscopic portal vein invasion	
Absent/present	198/36
Macroscopic hepatic vein invasion	
Absent/present	222/12
Microscopic portal vein invasion	
Absent/present	177/57
Microscopic hepatic vein invasion	
Absent/present	181/53
AFP (ng/ml)	
<400/≥400	179/55
Differentiation	
Well/moderate/poor	46/153/35
Anatomical resection	
Yes/no	159/75
Staging systems	
TNM stage (LCSGJ)	
I/II/III/IV	25/87/85/37
TNM stage (UICC)	
I/II/III/IV	104/103/25/2
JIS score	
0/1/2/3–4	20/84/81/49
Modified JIS score	
0/1/2/3–4	16/63/88/67
CLIP	
0/1/2/3–5	109/78/32/15
Tokyo score	
0/1/2/3/4–6	14/79/81/42/18

AFP, α-fetoprotein; CLIP, Cancer of the Liver Italian Program; HBV, hepatitis B virus; HCV, hepatitis C virus; ICG-R15, Indocyanine green retention rate at 15 min; JIS, Japan Integrated Staging score; LCSGJ, Liver Cancer Study Group of Japan; non-BC, patients negative for both HBS antigen and HCV antibody; NxS, number and size; TNM, tumor lymph node metastasis; UICC, Union for International Cancer Control.

**Table II:** Univariate analysis (log-rank test) and multivariate analysis (Cox proportional hazards model) of variables potentially predictive of disease-free survival in patients with HCC (n=234).

	Univariate analysis		Multivariate analysis	
		
Variable	Median survival (95% CI) (months)	P-value	HR (95% CI)	P-value
NxS factor		<0.0001		
<4	42.3 (26.4–63.3)		1	
4–9	20.5 (15.4–33.1)		1.43 (1.02–2.00)	0.0364
>9	15.5 (8.1–18.2)		2.77 (1.83–4.17)	<0.0001
Microscopic portal vein invasion		0.0015		
Absent	29.6 (21.9–39.3)		1	
Present	17.5 (9.4–22.9)		1.31 (0.91–1.86)	0.1464
Microscopic hepatic vein invasion		0.0072		
Absent	26.4 (21.0–36.5)		1	
Present	17.5 (9.3–23.5)		1.35 (0.92–1.94)	0.1237
Differentiation		0.3236		
Well	32.4 (22.5–56.1)			
Moderate	21.0 (18.2–26.4)			
Poor	22.9 (15.0–41.6)			
AFP		0.7067		
<400	24.4 (20.2–31.3)			
>400	19.2 (7.3–35.8)			
Child-Pugh		0.0281		
A	24.1 (20.6–30.0)		1	
B	18.5 (9.2–36.5)		1.28 (0.79–2.04)	0.3026
Degree of liver damage		0.0002		
A	30.4 (22.4–43.6)		1	
B	18.9 (14.9–21.9)		1.93 (1.37–2.70)	0.0002

HCC, hepatocellular carcinoma; CI, confidence interval; HR, hazard ratio; AFP, α-fetoprotein.

**Table III:** PS score.

	Score		
	
	0	1	2
NxS factor	<4	4–9	>9
Liver damage	A	B	

NxS, number and size; PS, predictive staging.

**Table IV:** Comparison of prognostic stratification.

	Component of the score		AIC	
		
Model	Tumor factor	Liver function	DFS	OS
PS score	(+)	(+)	1791.19	1231.40
TNM (LCSGJ)	(+)	(−)	1810.51	1250.48
TNM (UICC)	(+)	(−)	1812.06	1242.38
JIS	(+)	(+)	1805.09	1249.85
m-JIS	(+)	(+)	1797.13	1240.21
CLIP	(+)	(+)	1816.89	1256.92
Tokyo	(+)	(+)	1811.63	1232.70

AIC; Akaike information criterion; CLIP, Cancer of the Liver Italian Program; DFS, disease-free survival; JIS, Japan Integrated Staging score; LCSGJ, Liver Cancer Study Group of Japan; m-JIS, modified Japan Integrated Staging score; OS, overall survival; PS, predictive staging; UICC, Union for International Cancer Control.
